# Placentation in the anteaters *Myrmecophaga tridactyla* and *Tamandua tetradactyla* (Eutheria, Xenarthra)

**DOI:** 10.1186/1477-7827-10-102

**Published:** 2012-11-30

**Authors:** Andrea M Mess, Phelipe O Favaron, Christiane Pfarrer, Christine Osmann, Allan PF Melo, Rosangela F Rodrigues, Carlos E Ambrósio, Estela Bevilacqua, Maria A Miglino

**Affiliations:** 1 Department of Surgery, Faculty of Veterinary Medicine and Animal Science, University of Sao Paulo, Av. Prof. Dr. Orlando Marques de Paiva, 87, Cidade Universitária, São Paulo, SP, CEP 05508-270, Brazil; 2 Institute of Anatomy, University of Veterinary Medicine Hannover, Bischofsholer Damm 15, 30173, Hannover, Germany; 3 Zoo Dortmund, Mergelteichstr. 80, 44225, Dortmund, Germany; 4 Department of Basic Science, Faculty of Animal Sciences and Food Engineering, University of Sao Paulo, Av. Duque de Caxias Norte, 225, ZAB, Pirassununga, CEP 13635-900, Brazil; 5 Institute of Biomedical Sciences, University of Sao Paulo, Av. Prof. Lineu Prestes, 1524, Cidade Universitária, São Paulo, SP, CEP 05508-900, Brazil

**Keywords:** Evolution, Vermilingua, Trophoblast, Interhaemal barrier, Villous placenta

## Abstract

**Background:**

Since Xenarthra are serious candidates for being basal to Eutheria, their characteristics, e.g. the placental system, influence perceptions of evolution. However, in the subgroup containing the anteaters, data are very limited. The present study aims to elucidate the nature of the feto-maternal interface in the anteater placenta and to interpret these data within an evolutionary context.

**Methods:**

Placentas of two species were investigated with histology, immunohistochemistry and transmission electron microscopy.

**Results:**

Remnants of the maternal vessel endothelium were absent, resulting in a fully haemochorial barrier throughout the placenta. Two structurally different parts, the villous and trabecular areas were complex and intermingled. In particular, the trabeculae which consisted of cellular, proliferative trophoblast, associated with connective tissue, were attached to the decidua. The villi contained fetal capillaries and hypertrophied mesenchymal cells that occured near the surface near the end of gestation. The surface of the villi consisted of flat, syncytial trophoblast, interspersed with proliferative trophoblast cells.

**Conclusions:**

Based on fundamental differences between anteaters and armadillos, we inferred that placental evolution was more complex than previously thought. The haemochorial pattern of anteaters was likely an ancient condition of xenarthrans. Consequently, villous placentation may be attributed, at least in part, by convergent evolution, but was also characterized by some features that were widespread among xenarthrans.

## Background

Xenarthra is a group of eutherian mammals that evolved in South America since the mid Paleocene and subsequently radiated successfully [1-3]. Three distinct groups evolved: Cingulata comprises armadillos (Dasypodidae), Pilosa include sloths (Bradypodidae and Megalonychidae) and anteaters (Vermilingua: Myrmecophagidae and Cyclopedidae) [4-6]. The latter had a specialized, elongated rostrum, prominent claws and long gestation periods; they were solitary, crepuscular and inhabited grasslands and other habitats of Central South America [7-10]. Xenarthra represents a supraordinal clade of Eutheria. Since they are serious candidates for being basal to Eutheria [11-13], their character conditions influence perceptions of eutherian evolution [14]. In particular, placental characters vary among xenarthrans [15-19]. Placentation has been well characterized in armadillos [20-26]; they have villous and haemochorial placentas formed by a peculiar, partly invasive interaction with maternal vessels [25-27]. In contrast sloths have lobulated, labyrinthine and endotheliochorial placentas [28-30]. Anteaters are regarded as being similar to armadillos. Consequently, an armadillo-like pattern is regarded to represent the ancient condition of Xenarthra, resulting in evolutionary transformations on the stem lineage of sloths [31]. However, data on anteater placentation are limited to an early stage [32] and delivered placentas [33] of the giant anteater *Myrmecophaga tridactyla*, approximately 10 stages from early- to mid-gestation of the lesser anteater *Tamandua tetradactyla*[34] as well as a single, late stage of the two-toed anteater *Cyclopes didactyla*[35]. Important aspects are unresolved, i.e. the degree of trophoblast invasion, development and fine structure of the trabecular area, contribution of fetal or maternal tissues to them as well as the presence or absence of cellular trophoblast in the villi at term [33]. The objective of the present study was to use histology, immunohistochemistry and transmission electron microscopy to characterize similarities and differences among xenarthrans and to interpret these data in an evolutionary context.

## Methods

### Tissue collection

Material from *Myrmecophaga tridactyla*, acquired from a road-killed animal in Brazil, represented mid gestation (approximately 100–110 days [36,37]). Three delivered placentas were obtained from the breeding group at Dortmund Zoo, Germany. A near-term stage from *Tamandua tetradactyla* that was more advanced than those described by Becher [34] was derived from the zoological park in Ilha Solteira, Brazil. This research was approved by the Ethical Committee at the Faculty of Veterinary Medicine and Animal Science of the University of Sao Paulo.

### Histology and immunohistochemistry

Material for histology, fixed in 10% formalin in 0.1 M phosphate buffer or Bouin’s solution, was embedded in paraplast, sectioned at 5 μm in an automatic microtome (Leica RM 2155, Nussloch, Germany), and stained with haematoxylin and eosin, Masson’s trichrome, toluidine blue and the periodic acid Schiff reaction (PAS). Immunohistochemistry (for details see [26,38]) for vimentin was done to detect mesenchymal cells, including remnants of the maternal endothelium and stromal decidua (mouse monoclonal anti-human antibody; RTU-VimV9; 1:300; Novacastra; Wetzlar, Germany), α-smooth muscle actin that similarly labeled vessel walls (1:400; Clone 1A4; Dako Cytomation; Carpinteria, California, USA), cytokeratin to identify epithelial tissues including trophoblast (rabbit polyclonal antibody; wide spectrum screening N1512; 1:100; Dako) and as proliferation marker a mouse monoclonal antibody to human anti-PCNA (proliferation cell nuclear antigen; clone PC10; 1:300; Sigma; St. Louis, USA). Sections were subjected to endogenous peroxidase blockage, non-specific binding was blocked [38], incubated with the primary antibodies overnight at 4°C in a humid chamber, and rinsed in PBS. A biotinylated secondary antibody and streptavidin-HRP (Dako) were applied for 30 min each, followed by rinsing with PBS. Detection was done with Fast Red TR/Naphthol AS-MX (F4523, Sigma) or DAB and substrate chromogen system (Dako) for 2 min, counterstained with haematoxylin and eosin and mounted in Faramont® (Dako). Negative controls used a goat anti-Mouse IgG (AP308F, 1:500;Chemicon International Temecula, California, USA) in lieu of primary antibody. Slides were examined with an Olympus BX40 microscope with Zeiss KS400 image analysis system.

### Transmission electron microscopy

Samples for TEM were fixed in 2.5% glutaraldehyde in cacodylate buffer, post-fixed in 2% phosphate-buffered osmium tetroxide at ph 7.4 for 2 h, embedded in Spurr’s Resin and sectioned with an automatic ultramicrotome (Ultracut R, Leica). Semi-thin sections (400 nm) were stained with toluidine blue. Ultrathin sections (90 nm) were contrasted with 2% uranyl acetate and 0.5% lead citrate and studied in an electron microscope (Morgagni 268D, FEI Company, The Netherlands; Mega View III camera, Soft Imaging System, Germany).

## Results

### Myrmecophaga tridactyla: mid gestation placenta

**Figure 1:**
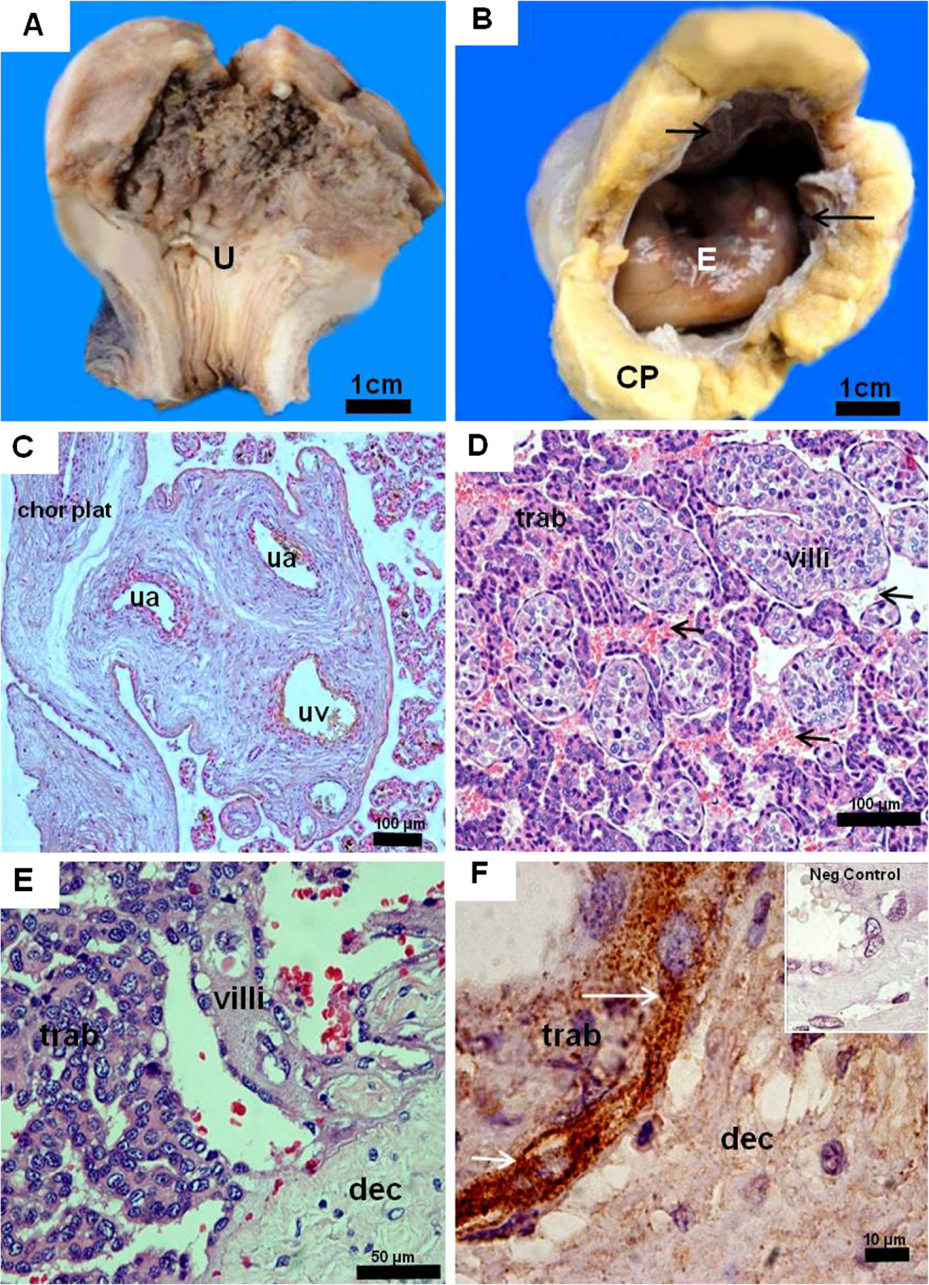
***Myrmecophaga tridactyla *, mid gestation placenta.** (**A**,**B**) Macroscopic anatomy. Uterus (U) with lobulated structure, an extended chorioallantoic placenta (CP), areas of gelatinous appearance (arrows), and a single embryo (E). (**C**) Haematoxylin and eosin. One umbilical vein (uv) and two arteries (ua) entered the placenta from the chorionic plate (chor plat). (**D**) Haematoxylin and eosin. Intermingling of villous (villi) and trabeculae (trab) areas, bathed in maternal blood (arrows). (**E**) Haematoxylin and eosin. Both villi and trabeculae reached the decidua (dec). (**F**) Cytokeratin-positive trophoblast of trabeculae (arrows) attached to the decidua that was cytokeratin-negative.

**Figure 2:**
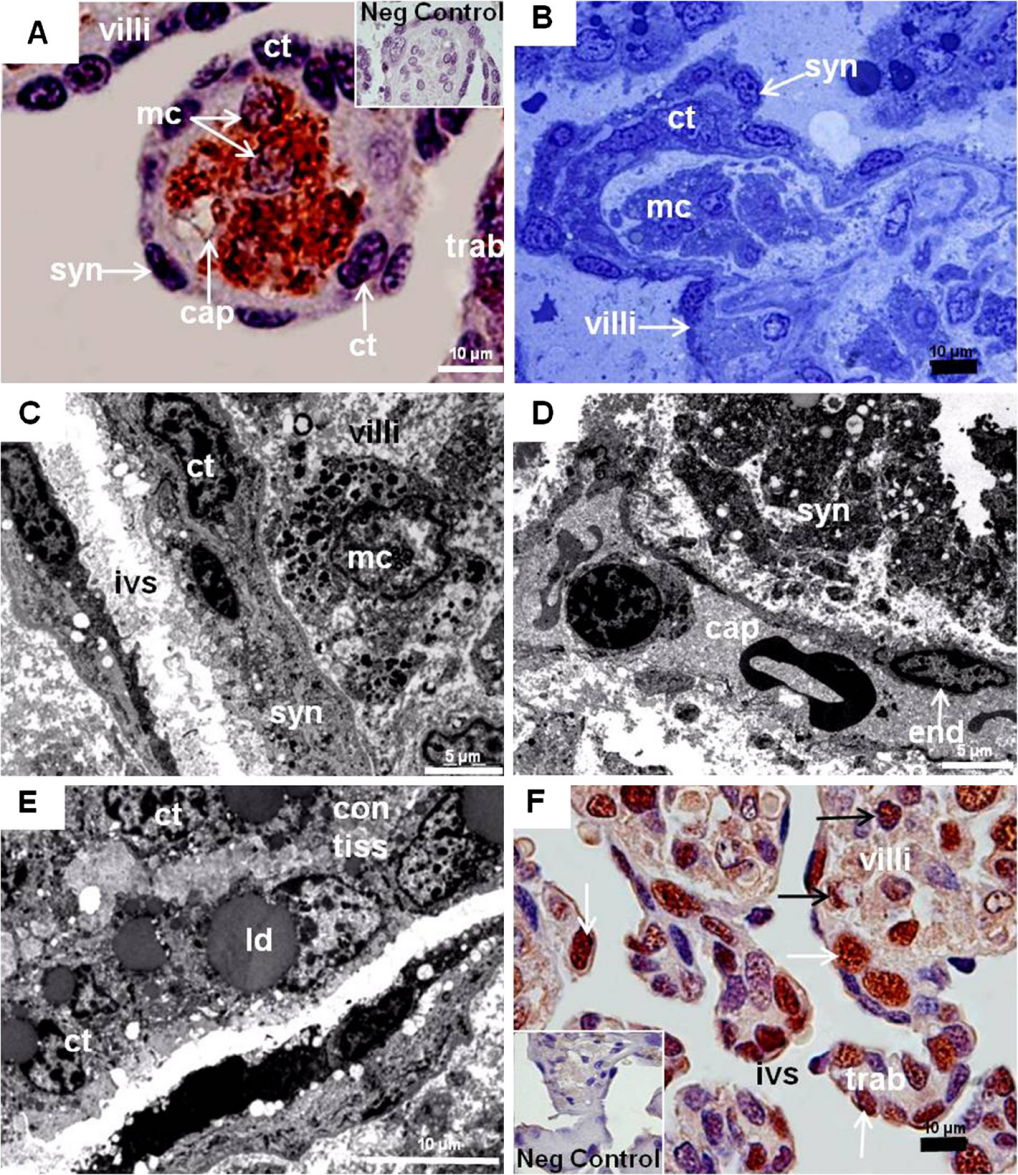
***Myrmecophaga tridactyla *, mid gestation placenta.** (**A**) Vimentin. Villi with positive connective tissue, enlarged mesenchymal cells (mc) and fetal capillaries (cap). Cellular (ct) and syncytial (syn) trophoblast were immunonegative, as well as trophoblast of trabeculae (trab). Remnants of the maternal vessel endothelium were absent. (**B**) Toluidine blue. Villi with two layers of trophoblast and hypertrophied mesenchymal cells. (**C**,**D**) TEM. The interhaemal barrier along the intervillous space (ivs) was thin and syncytial. Trophoblast cells occurred. Fetal capillaries with endothelium (endo) were near the surface (**E**) TEM. Trabeculae had solid strands of cellular trophoblast with large nuclei and liquid droplets (ld) and connective tissue (con tiss). (**F**) PCNA. Proliferation activity was high in trophoblast cell clusters of villi and trabeculae (white arrows). Also, proliferation occurred in hypertrophied mesenchymal cells and endothelia of the villi (black arrows).

The CRL was 7 cm. The extended placenta was approximately 6 cm in diameter and 1 to 1.5 cm thick. The conceptus occupied most of the uterine cavity, located at the fundic area, which was lobulated (Figure 1A). The surface of the conceptus was gelatinous and opaque (Figure 1B). In the amnion, vascularisation had begun and fibrinoid plaques were present. The umbilical cord contained one umbilical vein and two arteries that entered the placenta (Figure 1C). The latter consisted of villous and trabecular areas, organized in lobes (Figure 1D). Both areas were intermingled and reached the decidua (Figure 1D,E), but were mostly separate (Figure 1F). Remnants of maternal vessel endothelium were absent along the villi and trabeculae (Figure 2A). The villi were complex (Figure 1D). Their surface was formed by trophoblast (Figure 2A,B). An outer layer was syncytial (Figure 2C,D), accompanied by an inner, locally discontinuous layer of cytotrophoblast (Figure 2B,C). The barrier was thin in parts (Figure 2D). Inside the villi, connective tissue, hypertrophied mesenchymal cells and capillaries were present (Figure 2A-D). Occasionally, capillaries were near the surface (Figure 2D). The villi were confluent with the trabeculae (Figure 2A), which consisted of cellular trophoblast with large nuclei and liquid droplets, sourrounded by connective tissue (Figure 2A,E). The cellular trophoblast of both the villi and trabeculae was proliferative (Figure 2F); the tips of the villi were particularly active. In addition, mesenchymal cells and capillary endothelium were positive (Figure 2F).

### Myrmecophaga tridactyla: term placentas

**Figure 3:**
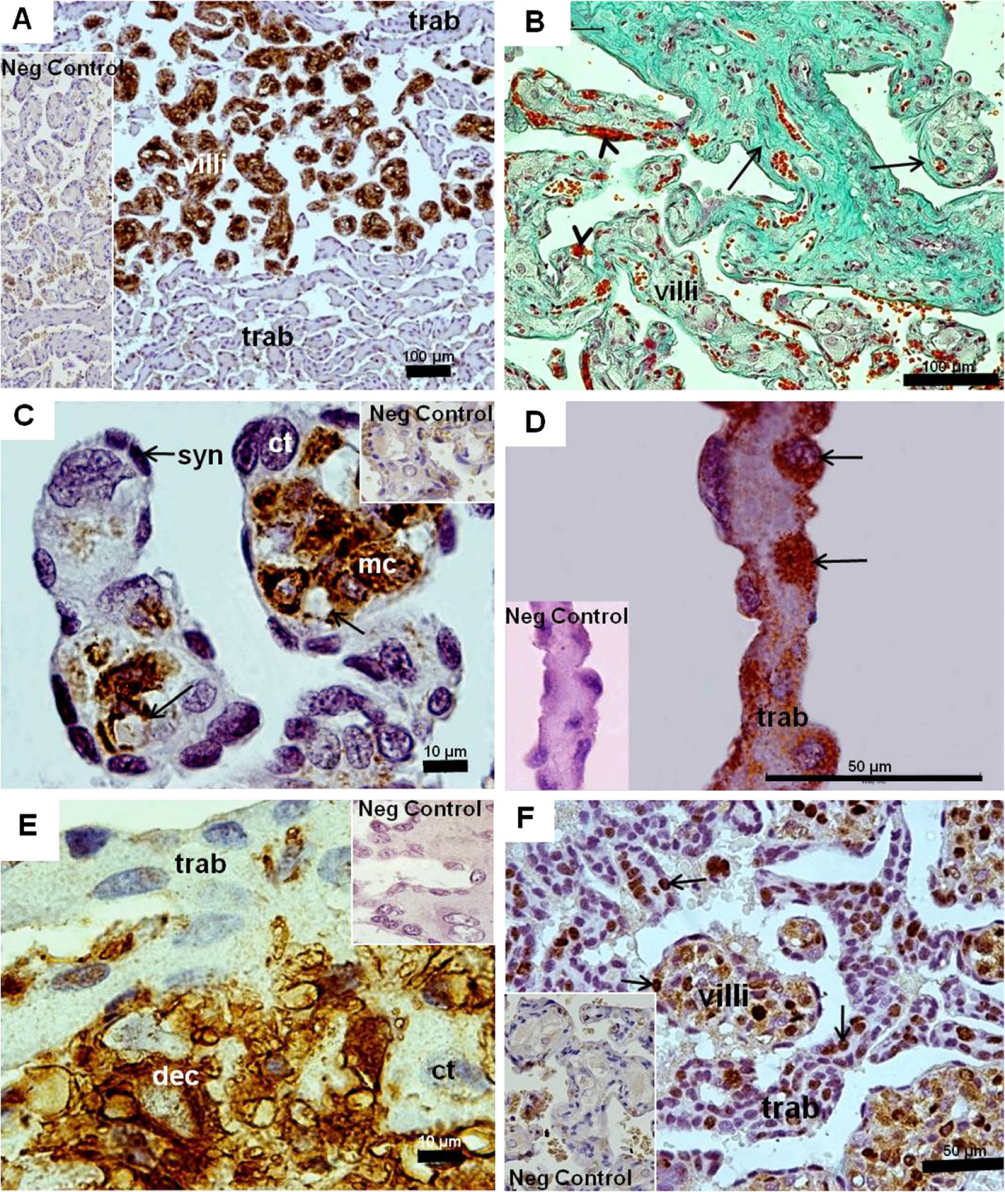
***Myrmecophaga tridactyla*, term placentas.** (**A**) Vimentin. Villi were strongly vimentin-positive, in contrast to the solid trophoblast of trabeculae (trab). (**B**) Masson’s trichrome. Branching villi (arrows) with fibers. Fetal capillaries (arrowheads) near the surface of the villi. (**C**) Vimentin-negative syncytial trophoblast (syn) and trophoblast cells (ct). Positive response in hypertrophied mesenchymal cells (mc) and capillary endothelium (arrow). No remnants of the maternal endothelium were present along the villi and trabeculae. (**D**) Cytokeratin-positive trophoblast of the trabeculae (arrow). (**E**) Vimentin. Trophoblast (ct) of trabeculae (vimentin negative) occasionally invaded decidua (vimentin-positive). (**F**) PCNA. Proliferation in trophoblast of villi and trabeculae (arrows).

All term placentas were discoidal. The umbilical cord was prominent, with one vein and two arteries. Inside the disc, both villous and trabecular areas were present (Figure 3A). The decidua was thin. On comparison to mid-gestation, the villous region had increased complexity and volume. The projections of the villi were intermingled with the trophoblast of the trabeculae, but did not reach the decidua (Figure 3A). The villi had abundant fibers, connective tissue and enlarged mesenchymal cells, and were well vascularized (Figure 3B,C). The capillaries were near the surface (Figure 3C). The trophoblastic surface of the villi was syncytial and thin; however, there were single trophoblast cells towards the interior (Figure 3C). The trabeculae consisted of cellular trophoblast with limited syncytial areas and connective tissue (Figure 3D). At the placental base, the trabeculae were near the decidua, but only occasionally trophoblast cells invaded the surface (Figure 3E). The trophoblast cells in the tips of the villi and the trabeculae were proliferating (Figure 3F).

### Tamandua tetradactyla: late gestation or near-term placenta

**Figure 4:**
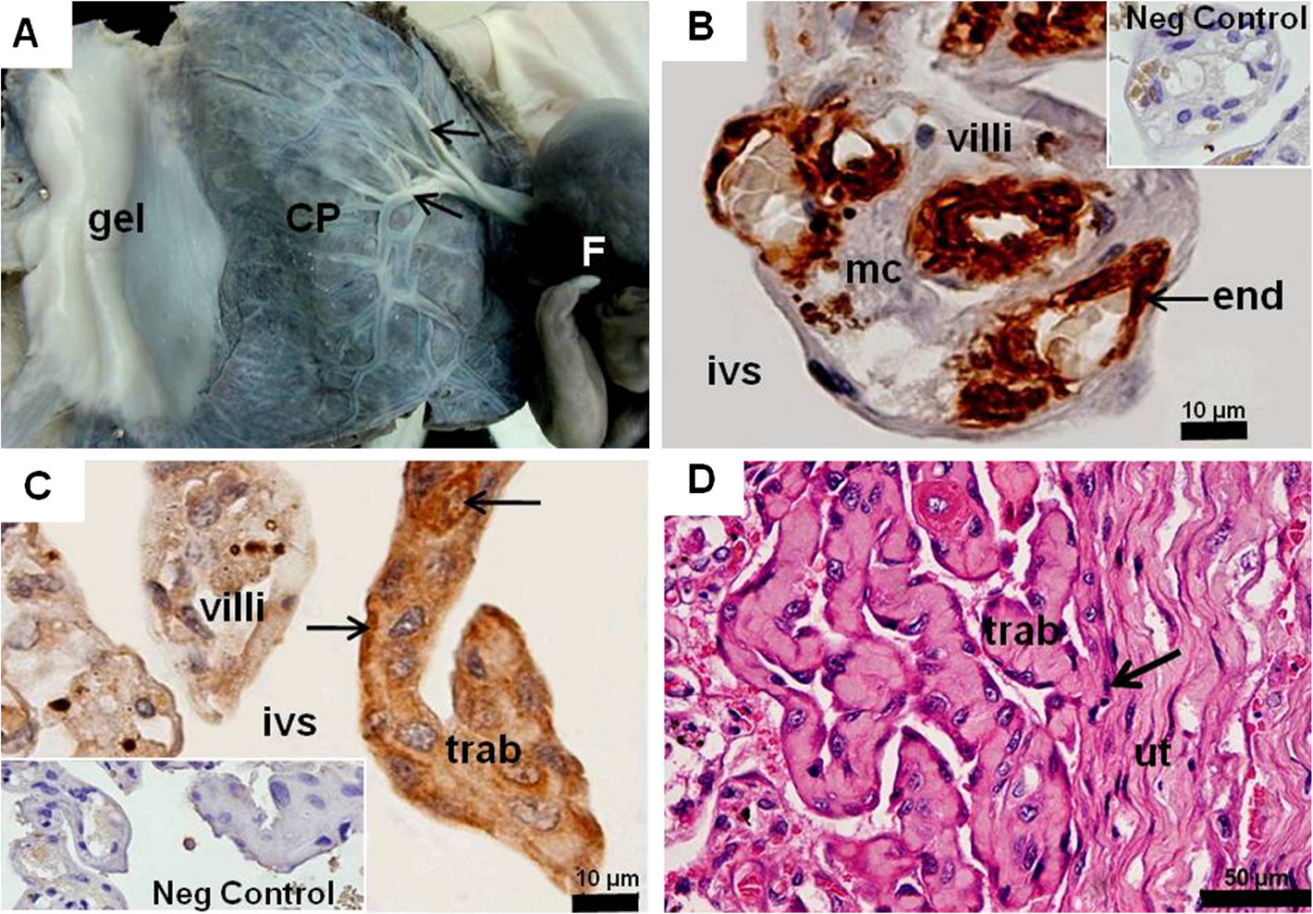
***Tamandua tetradactyla *, late gestation or near term placenta.** (**A**) Macroscopic anatomy. Placenta disc (CP) with branching umbilical vessels (arrows) along an area of gelatinous tissue (gel) and a single fetus (F). (**B**) α-smooth muscle actin. Villi in the intervillous space (ivs) with immuno-positive fetal capillary endothelium (end) and hypertrophied mesenchymal cells (mc). No remnants of maternal endothelium were present. (**C**) Cytokeratin. Trophoblast cells of the trabeculae (trab) were positive (arrows), facing towards the intervillous space. (**D**) Haematoxylin and eosin. Trophoblast of the trabeculae (arrow) attached to the connective tissue and musculature of the uterus (ut).

The CRL was approximately 12 cm and the discoidal placenta was 10 cm in diameter (Figure 4A). The conceptus occupied approximately 75% of the fundic area of the uterine cavity. Gelatinous tissue covered the surface of the conceptus (Figure 4A). A thin amniotic membrane covered the placenta. The umbilical cord was 11 cm. It contained one umbilical vein and two arteries that complexly branched at the chorionic plate (Figure 4A). The placenta was organized into lobes. The villi were intermingled with trabeculae. Remnants of maternal endothelium were absent (Figure 4B,C). Villi were lined by syncytiotrophoblast with some cytotrophoblast and contained connective tissue, fetal capillaries, and hypertrophied mesenchymal cells. Capillaries were near the surface (Figure 4B). The villi were connected to the trabeculae that consisted of trophoblast cells with round nuclei and connective tissue inside (Figure 4C). The trophoblast cells were proliferative. Both villi and trabeculae reached the decidua and were close, but remaining distinct (Figure 4D).

## Discussion

Major aspects of placentation fundamentally differed between anteaters and armadillos. Consequently, an armadillo-like pattern could not be regarded as an ancient condition of xenarthrans. In particular, the feto-maternal interface in anteaters was haemochorial without remnants of the maternal vessel endothelium along the trabeculae. Confirmation derived by application of immunohistochemistry and transmission electron microscopy supported previous hypotheses that during early gestation, the trophoblast is fully invasive throughout the placenta [32,34], independent of a sinusoid area of maternal vessels present deep inside the uterus in an early stage of *Tamandua*[34]. In contrast, developing villi in armadillos entered maternal blood sinuses and enlarged them, leaving the endothelium largely intact. Fetal tissues developed *inside* these sinuses [25]. Since anteaters and armadillos differed from each other as well as in comparison to sloths that had an endotheliochorial type of the barrier [28-30]), the evolutionary courses of placentation among xenarthrans were difficult to establish. However, the pattern manifest in anteaters likely represented an ancient condition, because haemochorial placentation was widespread in eutherian mammals, or may even belong to their stem species pattern [15-19]. Consequently, the unique pattern of haemochorial villous areas *and* endotheliochorial blood sinuses may be the result of evolutionary transformations on armadillo stem lineage.

Secondly, the depth of trophoblast invasion in anteaters was less than in armadillos. Trophoblast cells invaded the surface of the decidua; only in *Tamandua* were larger parts of the decidua resorbed (own results, [31-34]), but the fetal tissues did not reach deeply into the myometrium as in armadillos [20-26]. A relatively superficial invasion occurred also in sloths, where syncytial trophoblast was present along endometrial vessels [28-30]. Consequently, trophoblast invasion in the last common ancestor of xenarthrans seemed to be restricted to the endometrium and deciduas, whereas the very deep invasion in armadillos was attributed to a subsequent evolutionary transformation.

However, anteaters and armadillos also shared important similarities (own results, [20-27,32-35]), i.e. a placental establishment at the fundic region of the uterus, the relatively extended to disc-like placental shape, complex intermingled trabecular and villous areas, dominance of cellular trophoblast with some connective tissue in the trabeculae, syncytiotrophoblastic and partly thin surface of the villi with interspersed trophoblast cells towards the interior, the location of fetal capillaries near the surface of the villi in association to hypertrophied mesenchymal cells, and presence of proliferating trophoblast at the tips of the villi. Due to the widespread distribution of these features within xenarthrans, they likely represented ancient character conditions of the group.

## Conclusions

The present study addressed anteater placentation, namely trophoblast invasion and the nature of the feto-maternal interface, and provided new insights regarding the course of evolution of placental characters among xenarthrans. There were fundamental differences between anteaters and armadillos. In particular, maternal endothelium was completely absent throughout the placenta, which was restricted to the endometrium and decidua. This pattern was more likely to be ancient for xenarthrans than the unique mixture of haemochorial *and* endotheliochorial areas in armadillos. Thus, the villous placenta may have been established by convergent evolution. However, we identified a number of shared similarities that likely were part of the xenarthran stem species pattern. In conclusion, an armadillo-like pattern should not be regarded as ancient condition of xenarthrans, because their placental evolution was more complex than previously established.

## Competing interests

The authors declare that they have no competing interests.

## Authors' contributions

MAM devised the study and participated in its design. AMM analyzed the material and wrote the manuscript, supported by POF and CP. All other authors were involved in the acquisition and procession of this rare material. All authors read and approved the final manuscript.
